# Trends in Diet and Cancer Research: A Bibliometric and Visualization Analysis

**DOI:** 10.3390/cancers15153761

**Published:** 2023-07-25

**Authors:** Erin D. Giles, Sarah A. Purcell, Jessica Olson, Alina Vrieling, Kelly A. Hirko, Kary Woodruff, Mary C. Playdon, Gwendolyn A. Thomas, L. Anne Gilmore, Heather K. Moberly, Annie E. Newell-Fugate

**Affiliations:** 1 School of Kinesiology and Rogel Cancer Center, University of Michigan, Ann Arbor, MI 48109, USA; 2 Department of Medicine, Division of Endocrinology, University of British Columbia, Vancouver, BC V5Z 1M9, Canada; sarah.purcell@ubc.ca; 3 Department of Biology, Irving K. Barber Faculty of Science, University of British Columbia Okanagan, Kelowna, BC V1V 1V7, Canada; 4 Division of Community Health, Institute for Health & Equity, Medical College of Wisconsin, Milwaukee, WI 53226, USA; jeolson@mcw.edu; 5 Department for Health Evidence, Radboud University Medical Center, 6525 GA Nijmegen, The Netherlands; alina.vrieling@radboudumc.nl; 6 Department of Epidemiology and Biostatistics, College of Human Medicine, Michigan State University, East Lansing, MI 48825, USA; hirkokel@msu.edu; 7 Department of Nutrition and Integrative Physiology, University of Utah, Salt Lake City, UT 84112, USA; kary.woodruff@utah.edu; 8 Cancer Control and Population Sciences, Huntsman Cancer Institute, Salt Lake City, UT 84112, USA; mary.playdon@hci.utah.edu; 9 Department of Kinesiology, The Pennsylvania State University, University Park, PA 16802, USA; gat112@psu.edu; 10 Department of Clinical Nutrition, UT Southwestern Medical Center, Dallas, TX 75390, USA; linda.gilmore@utsouthwestern.edu; 11 University Libraries, The Pennsylvania State University, University Park, PA 16802, USA; hkm1@psu.edu; 12 Department of Veterinary Physiology & Pharmacology, School of Veterinary Medicine and Biomedical Sciences, Texas A&M University, College Station, TX 77843, USA

**Keywords:** dietary intake, nutritional status, cancer, neoplasms, bibliometrics, food intake

## Abstract

**Simple Summary:**

Diet plays an important role in modifying cancer risk and may improve outcomes for patients during and after cancer treatment. The goals of this bibliometric review were to characterize studies, highlight emerging trends, and identify gaps in the literature regarding diet and cancer. We found that while previous publications have focused on the impact of high-fat diets and alcohol on common cancers such as breast, colorectal, and liver, there are far fewer publications describing the role of diet in less prevalent cancers. Areas of emerging interest include studies on nutrient timing, spices, and pre- and probiotics.

**Abstract:**

Diet plays a critical role for patients across the cancer continuum. The World Cancer Research Fund International and the American Cancer Society have published evidence supporting the role of nutrition in cancer prevention. We conducted an analysis of the literature on dietary nutrients and cancer to uncover opportunities for future research. The objective of the bibliometric analysis was to describe trends in peer-reviewed publications on dietary components and cancer and to highlight research gaps. PubMed was queried for manuscripts with diet- and cancer-related keywords and Medical Subject Headings (MeSH) terms. Metadata covering 99,784 publications from 6469 journals were analyzed to identify trends since 1970 on diet topics across 19 tumor types. Publications focused largely on breast, colorectal, and liver cancer, with fewer papers linking diet with other cancers such as brain, gallbladder, or ovarian. With respect to “unhealthy” diets, many publications focused on high-fat diets and alcohol consumption. The largest numbers of publications related to “healthy” diets examined the Mediterranean diet and the consumption of fruits and vegetables. These findings highlight the need for additional research focused on under-investigated cancers and dietary components, as well as dietary studies during cancer therapy and post-therapy, which may help to prolong survivorship.

## 1. Introduction

Diet is a critical, modifiable behavior that affects cancer risk, treatment, and prognosis [1]. The World Cancer Research Fund (WCRF)/American Institute for Cancer Research (AICR) and the American Cancer Society (ACS) have published lifestyle guidelines for cancer prevention [1,2]. In the US, cancer survivors are strongly advised to follow these same recommendations [3]. The plethora of dietary recommendations include: maintaining a healthy weight; consuming primarily whole grains, fruits, vegetables, and legumes; limiting fast food, red meat, processed meat, sugary drinks, and alcohol; and avoiding dietary supplements unless medically indicated. Despite the strong evidence-based research that underlies these recommendations, the relationship between dietary nutrients and cancer prevention and survivorship is complicated. Many questions in this research space are yet to be resolved [4,5].

The WCRF/AICR and ACS reports provide a robust synthesis of the strong evidence in support of the role of diet in cancer prevention and survivorship [1,2]. However, a broad analysis of all available literature related to dietary patterns and nutrients and cancer onset or survivorship may uncover publication deficits and areas where additional research is needed. Bibliometric analyses offer a more refined approach to understand trends and reveal understudied areas in a specific research field by quantitatively assessing the scope of publications within a field, providing insights into trends, identifying areas with numerous publications, and highlighting gaps in the published literature [6]. Bibliometric analyses describe the characteristics of publications but do not critically appraise or synthesize the content within publications [6]. Such analyses often describe publication performance (i.e., publication characteristics and citations) and map a subject area through analyses of co-citations and keywords to identify trends and knowledge gaps in a specific research field [6,7,8]. In this way, bibliometric analyses are distinct from other methodologies, such as systematic reviews and meta-analyses, which focus on the critical appraisal of publication content and synthesize this content to answer highly focused but less generalizable questions [6,7,8]. Bibliometric analyses also provide insight into research trends over time, which are particularly relevant in understanding how dietary nutrients affect both cancer prevention and survivorship in the context of new dietary strategies popularized in the last decade (i.e., fasting, low-carbohydrate/ketogenic diets).

Within the cancer field, a PubMed search of “bibliometrics” and “neoplasms” (MeSH terms) indicates that more than 500 bibliometrics analyses have been conducted to date. Over the past ten years, these publications have focused on areas such as the gut microbiome [9], ketogenic diets [10], the Mediterranean diet [11], and specific cancers, including breast cancer [12]. There is also one bibliometric analysis of ~1500 papers related to cancer and nutrition published from 2011 to 2021, but this paper used only keywords that contained “nutrition”, rather than a more detailed interrogation of specific dietary components [13]. To our knowledge, no broad bibliometric analyses of diet types, dietary nutrients, or ingredients as well as cancer prevention and survivorship have been performed. Thus, the objective of this bibliometric analysis was to identify, quantify, and describe publication characteristics and trends in dietary components and cancer research and highlight gaps in the peer-reviewed literature in PubMed.

## 2. Materials and Methods

### 2.1. Databases and Search Strategy

PubMed was chosen as the source of bibliometric data for this analysis, because this database comprises more than 34 million citations and is available at no cost via the internet [14]. Medline, the largest component of PubMed, includes standardized terminology (i.e., MeSH terms) to describe each article [15].

To identify relevant publications on the broad topic of diet and cancer, we conducted trial searches in PubMed and the query terms were refined until the results included only publications relevant to the study. To do so, the title and abstract of the first 100 articles from each search were reviewed by the study team to assess the percentage of results directly related to the field of diet and cancer. The query was revised a total of 17 times, resulting in a search strategy that covered our topic area as broadly as possible but minimized unrelated hits. For example, the word “carb” could not be used as a search term without several exclusions, such as “carbon” and all other chemical derivatives of carbon that begin with these letters. During the revision process, the precision of the search strategy was improved by using official MeSH terms wherever possible. As an example, “cancer [MeSH]” was used in the trial searches. However, “cancer” is not an official MeSH term and, when searched in PubMed, the programming automatically maps it to the official MeSH term “neoplasms”. Similarly, “nutrition [MeSH]” was used in the trial searches and it automatically maps to “nutritional status [MeSH]”. Given this nuance of the PubMed algorithm, official MeSH terms were used so that future searches can replicate the results even if the underlying PubMed search algorithm programming changes. We chose not to include obesity or related terms in our search. While obesity may be a consequence of overnutrition (i.e., excess caloric intake relative to caloric needs for weight maintenance), our goal was to focus on studies of nutrients, food components, and dietary patterns.

### 2.2. PubMed Search Query

Once our search query was finalized in PubMed’s native interface, we used the pubmedR package for R (Version 0.0.3, available at http://github.com/massimoaria/pubmedR (accessed on 29 August 2022)), to repeat the search and pull the associated data from the PubMed database using NCBI REST APIs in the RStudio platform (https://rstudio.org (accessed on 29 August 2022)). Data were extracted on 29 August 2022. Our search query included diet and cancer terms in the title, abstract, and MeSH fields as follows:

(“diet”[MeSH Terms] OR “nutritional status”[MeSH Terms] OR “diet”[Title/Abstract] OR “diets”[Title/Abstract] OR “dietary”[Title/Abstract] OR “nutrition*”[Title/Abstract]) AND (“neoplasms”[MeSH Terms] OR “cancer*”[Title/Abstract] OR “tumor*”[Title/Abstract] OR “tumour*”[Title/Abstract]).

Search results (*n* = 101,692 publications) were downloaded in XML format in RStudio and the xml-structured object was converted to a classical data frame using the bibliometrix package for R (https://bibliometrix.org/ (accessed on 29 August 2022)). Since the purpose of a bibliometric review is to provide qualitative and quantitative insights into the development of research interests and collaborations over time to help anticipate emerging trends and address knowledge gaps, our search terms intentionally were broad. In keeping with our goal to broadly assess the literature on diet and cancer, only two filters were applied to the data before subsequent analyses. First, we removed papers published prior to 1970, to coincide with the establishment of the US National Cancer Act in 1971. Then, we removed an additional 399 items where the “publication type” included one of the following: address, autobiography, bibliography, biography, comment, congress, consensus development conference (NIH), dataset, directory, duplicate publications, interview, introductory journal article, lecture, newspaper article, overall, patient education handout, preprint. We did not limit the article language in the search as most papers had abstracts written in English, thus allowing us to search the abstracts regardless of publication language.

### 2.3. Data Analysis

The metadata were analyzed to identify the number of publications per annum by cancer type and keywords. For cancer type, we included the 17 cancers identified by the WCRF as diet- and/or physical activity-related (https://www.wcrf.org/diet-activity-and-cancer/cancer-types/ (accessed on 29 August 2022)). In addition, we also included brain cancers and leukemia, which have been frequently studied with respect to diet [16,17,18], for a total of 19 cancer types (Supplemental Table S1). For each cancer type, we searched in the MeSH field for the main cancer name, which was a combination of the organ or tissue type and “neoplasms” for all cancer types except leukemia. This MeSH search field term was combined with the more specific and descriptive terms searched in the title and abstract (i.e., organ or tissue combined with one of the following terms: “cancer”, “tumor”, “tumour”, “neoplasm”, or other terms depending on the cancer type).

We further searched the abstract field of each publication to assess diet-specific trends over time and by cancer type. These analyses included common dietary patterns and components and diet-related nutrients/supplements/vitamins. Dietary pattern terms mined from the titles and abstracts of publications using specific suites of search terms included caloric restriction, ketogenic/low-carbohydrate, high-fat, low-fat, Dietary Approaches to Stop Hypertension (DASH) diet, gluten-free, healthy diet patterns, Mediterranean diet, paleolithic, plant-based, time-restricted, and western diet. Dietary components that were searched from the main query were chosen based on their inclusion in the MeSH for “food” and the authors’ knowledge of the diet and cancer prevention and survivorship research literature (Supplemental Table S3). The search terms included in the assessment of dietary nutrients/supplements/vitamins originated from the combination of the MeSH for “supplements”, “nutrients”, and “vitamins” (Supplemental Table S4). Specific search terms are included in Supplemental Tables S2–S4; the selection of terms was informed by a review of the terminology from the previous literature and discussion amongst the study team. MeSH terms were included where possible. For each analysis, data are presented as trends over time and the total number of publications by cancer type.

### 2.4. Visual Network Analysis

Network analyses for MeSH keywords and author keywords were performed using the VOSviewer software (version 1.6.18) [19,20]. All analyses used the full counting method, in which each co-occurrence was given the same weight. To enhance figure readability, the maximum number of links in each figure was set to 200 and the minimum number of occurrences of a keyword was manually modified so that approximately 100 keywords would meet the chosen minimum threshold and 50 would appear in each figure. The minimum number of occurrences required for MeSH terms to meet this threshold was 1747 terms; the minimum number of occurrences for author-generated keywords was 148. VOSViewer builds upon modularity-based clustering by having the ability to detect small clusters of related items; clusters were manually named according to the most common and relevant keywords.

## 3. Results

### 3.1. Publications and Trends over Time

Our initial search identified 101,692 records in PubMed. The exclusion of articles based on publication year (*n* = 1509 before 1970) or record type (*n* = 399 not peer-reviewed scientific publications) resulted in 99,784 in 6228 journals. When the search for keywords/terms was restricted to the abstracts of papers in our database, it yielded a total of 91,249 papers containing variations of the words cancer, tumo(u)r, or neoplasia/neoplasm, or one of the specific cancer types (Supplemental Table S1); these publications were then used in all subsequent analyses. Most publications (83.2%) were classified as journal articles, 5.5% were clinical trials, 5.7% were comparative studies, 2.8% were conference/congress abstracts, and less than 1% were reviews, systematic reviews, and/or meta-analyses, with other document types (i.e., biographies, comments, editorials, evaluation studies) comprising the remaining publications. Within the figures, data over time are presented with cancer types or diet keywords with the highest prevalence first and the remainder in descending order.

**Figure 1:**
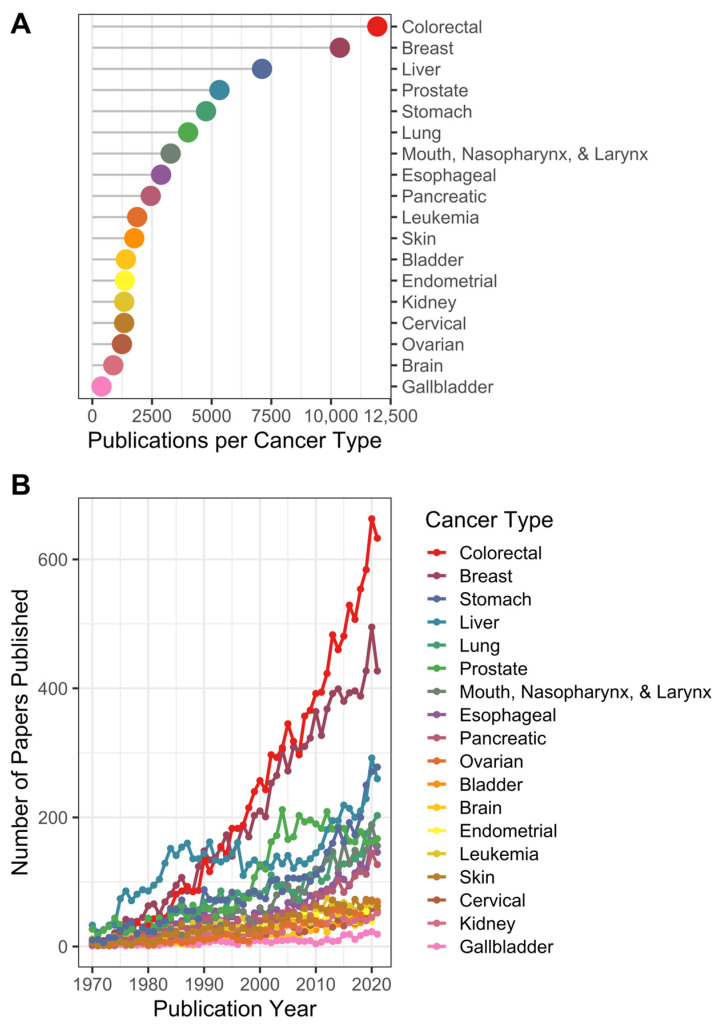
Total number of articles published per cancer type (1970–August 2022). Records meeting our diet–cancer search criteria were extracted from PubMed and were analyzed for (**A**) the total number of manuscripts containing cancer terms in the title or abstract and (**B**) numbers of manuscripts related to specific cancer types over time.

The annual growth rate of publications in this field over the study period was 5.15%. Of the total publications extracted from PubMed, only 91,249 specified one or more cancer types in the abstract, with colorectal (*n* = 11,942) and breast (*n* = 10,373) being the most highly referenced, followed by cancers of the liver (*n* = 7178), prostate (*n* = 5321), and stomach (4765); substantially fewer articles focused on other cancer types (Figure 1A; Supplemental Table S1). The number of articles published on diet and colorectal or diet and breast cancer has increased rapidly since 1980. Publications focused on diet and liver, prostate, and stomach cancer have also increased over this same period but at a much slower pace (Figure 1B). The number of publications on ovarian, bladder, brain, endometrial, skin, cervical, kidney, and gallbladder cancer, as well as leukemia, remained static over time and returned the lowest values (Figure 1B).

### 3.2. Diet Types, Components, and Bioactive Nutrient-Related Terms

**Figure 2:**
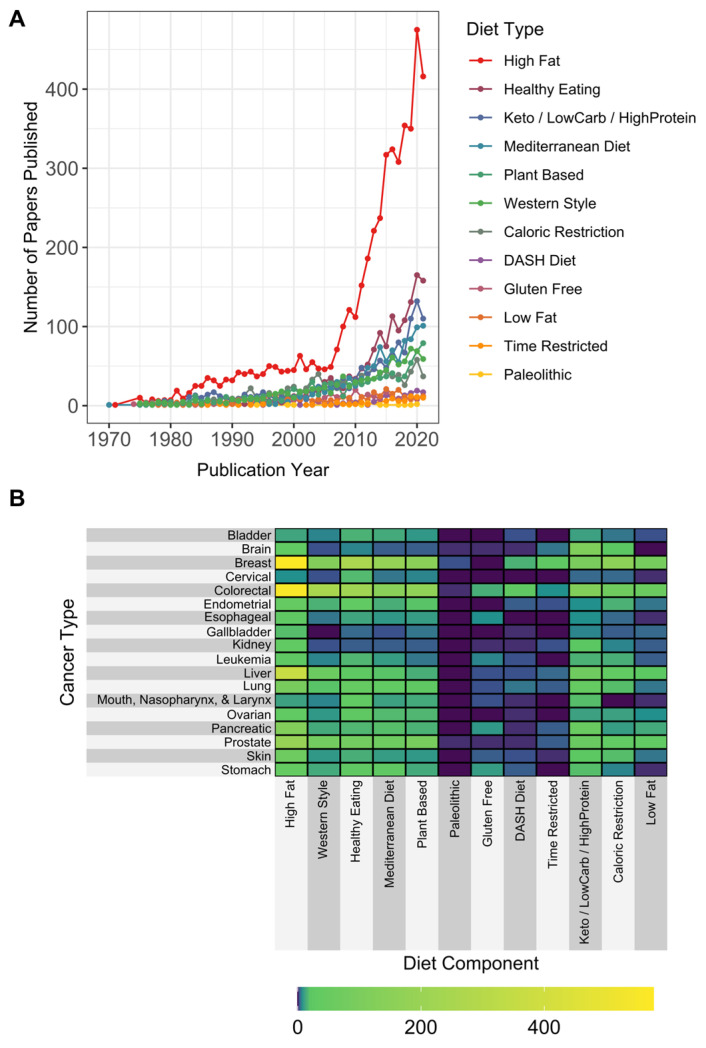
Number of papers published in the diet and cancer literature by diet type over time and by cancer type. Records were extracted from PubMed using the diet–cancer search and abstracts were analyzed for (**A**) specific diet types over time and (**B**) cumulative number of papers by diet and cancer type.

Trends in the number of papers published according to diet and cancer type over time are shown in Figure 2A,B, respectively, and the keyword/search terms associated with these data can be found in Supplemental Table S2. Breast, colorectal, and liver cancer had the greatest numbers of publications related to all diet types examined. Articles with terms in the abstract related to “high fat” constituted the largest number of results, with publications predominantly focused on breast, colorectal, liver, prostate, and pancreatic cancer (Figure 2A,B). Diet types with the fewest publications per cancer type were Dietary Approaches to Stop Hypertension (DASH), gluten-free, low-fat, time-restricted eating/feeding, and the paleolithic diet. Gluten-free diets were studied most frequently in colorectal, followed by stomach and pancreatic cancers. In addition to breast and colorectal cancer, brain cancer had the highest number of publications containing “keto/low-carb/high-protein” diet in the abstract.

**Figure 3:**
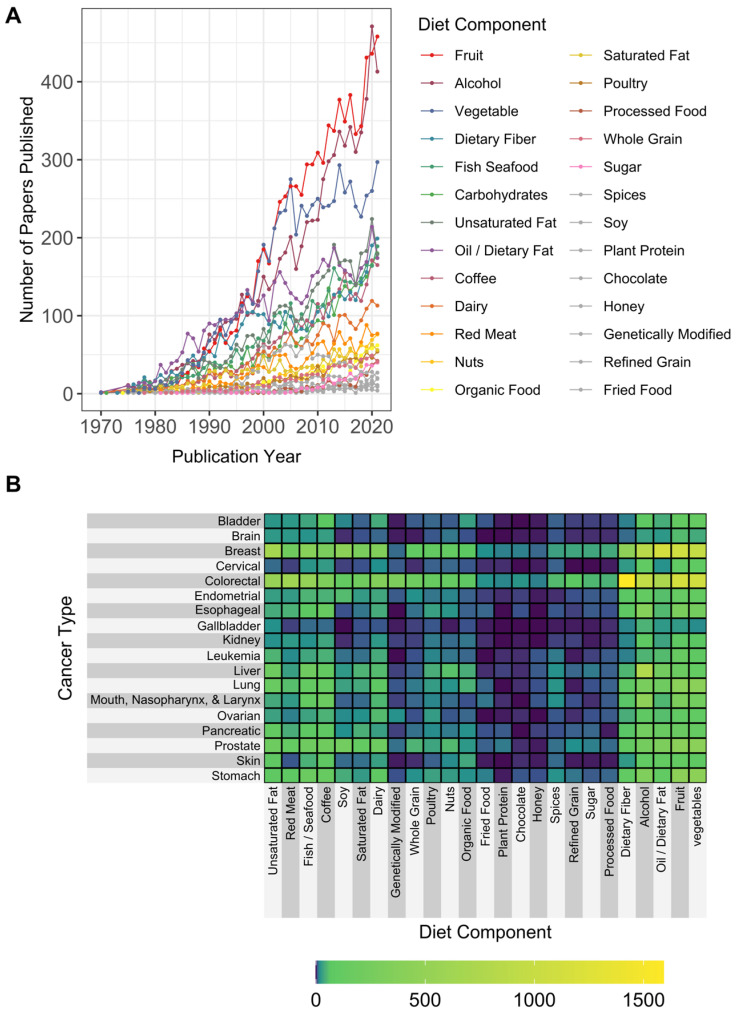
Individual dietary components assessed in the diet and cancer literature over time and by cancer type. Records were extracted from PubMed using the diet–cancer search and abstracts were analyzed for (**A**) specific dietary components over time and (**B**) cumulative number of papers by dietary component and cancer type.

Trends in the number of papers published according to dietary components and cancer types are displayed in Figure 3A,B, respectively, and the associated keyword/search terms are provided in Supplemental Table S3. Fruit and alcohol were the most common dietary components identified in the analysis of publications over time, with over 400 publications per annum in both 2021 and 2022. A rapid increase in the number of fruit- and alcohol-related publications occurred from 1990 through 2020, with a slight decline in the number of publications for alcohol from 2020 to 2021. Between 1970 and 2000, the number of publications focused on vegetables and cancer increased but has since leveled off. In contrast, the number of publications examining cancer and oil, fish/seafood, carbohydrates, fiber, sugar, saturated fat, dairy, red meat, nuts, organic food, poultry, or whole grains have increased consistently from 1970 through the present. As shown in Figure 3B, the largest numbers of publications by dietary component and cancer type were found for colorectal cancer, with studies focused on fiber, fruit, vegetables, alcohol, oil/dietary fat, and red meat. Breast cancer had the next largest number of publications related to dietary components, with studies on oil, fruit, vegetables, and alcohol. Overall, the smallest numbers of publications specific to cancer type were found for refined grains, chocolate, plant protein, fried food, and honey (Figure 3A). Additionally, few publications investigated dietary components in relation to cancers of the brain, cervix, gallbladder, or kidney compared to other cancer types.

**Figure 4:**
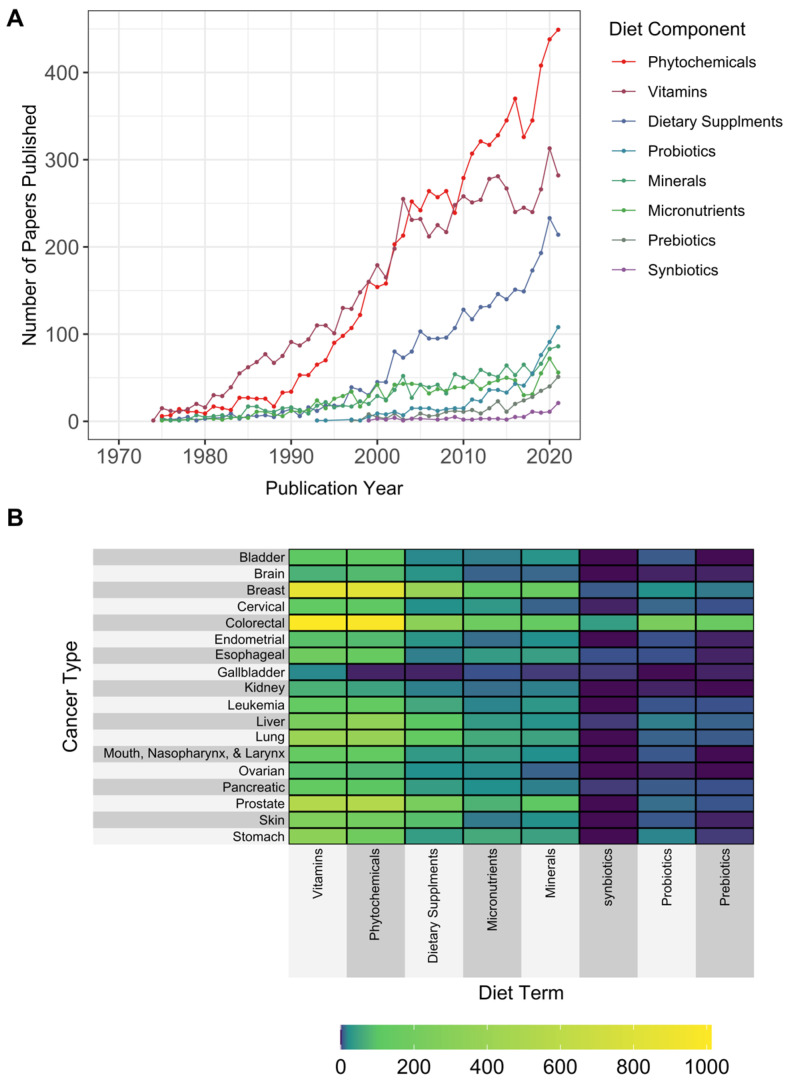
Distribution of publications on micronutrients and other bioactive components in relation to cancer types. Records were extracted from PubMed using the diet–cancer search and abstracts were analyzed for (**A**) specific micronutrients and bioactive components over time and (**B**) cumulative number of papers by micronutrient and bioactive component terms and cancer type.

The number of articles per cancer type and micronutrient or other bioactive components are presented in Figure 4 and associated keyword/search terms in Supplemental Table S4. A total of 20,731 publications were identified that included dietary nutrients/vitamins and cancer. Of this group, 12,931 publications focused on specific cancer types. Colorectal (*n* = 3059), breast (*n* = 2390), and prostate cancer (*n* = 1515) represented the greatest number of publications according to cancer type. The term “phytochemicals” generated the largest number of articles (*n* = 7432), with the most publications focused on colorectal and breast cancer relative to other cancer types. The term “vitamins” generated the second largest number of publications (*n* = 6742), followed by “dietary supplements” (*n* = 2924). The terms “prebiotics” and “probiotics” generated few publications (*n* = 346 and 701, respectively). “Prebiotics” and “probiotics” were used most frequently in publications studying colorectal cancer (*n* = 155 prebiotics and *n* = 229 probiotics).

### 3.3. Network Analysis of Keywords

**Figure 5:**
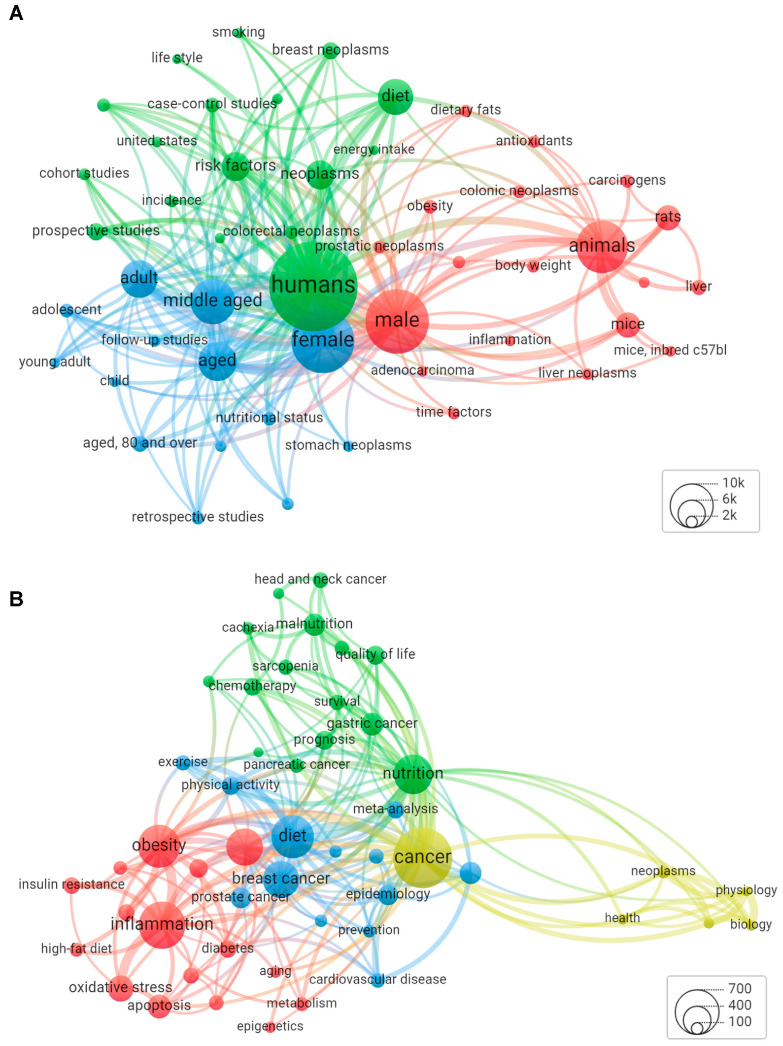
Network analysis of (**A**) MeSH keywords and (**B**) author keywords in articles focused on diet and cancer research. The top 50 keywords are shown. The size of each circle represents the number of papers with each keyword, while the thickness of the line represents the weight of connection between each topic.

Figure 5A,B show the co-occurrence network analyses of keywords, with the topic circle size representing the total volume of results (larger circles represent a greater number of results), lines representing connections among terms (thicker lines represent a greater number of articles using the same term), and the distance between terms representing term relatedness (closer distance represents stronger relatedness). MeSH-generated keywords created three clusters, in order of the volume of occurrence, as shown in Figure 5A (cluster group → largest nodes): (1) males, animals cellular mechanisms → males, animals, mice, and rats (red); (2) humans, lifestyle, risk → humans, diet, neoplasms, risk factors (green), and (4) female, age groups → female, middle-aged, aged, adult (blue); see Supplementary Figure S1. Author-generated keywords produced four clusters as shown in Figure 5B (cluster group → largest nodes): (1) inflammation, obesity, metabolic condition → inflammation, obesity, colorectal cancer, oxidative stress (red); (2) nutrition status, treatment, treatment outcomes → nutrition, gastric cancer, malnutrition, quality of life (green); (3) general lifestyle and study design terms → diet, breast cancer, mortality, prostate cancer (blue); and (5) general field terms → cancer, neoplasms, health, biology (yellow) (see Supplementary Figure S2). With MeSH-generated keywords, the strongest links were among terms that described the population (e.g., humans, animals, male, female, middle-aged, etc.). Among author-generated keywords, the strongest links were among those that described the condition or topic (e.g., cancer, nutrition, obesity, inflammation).

## 4. Discussion

### 4.1. Principal Findings

In this study, we systematically investigated and summarized the characteristics of research related to dietary components/nutrients and cancer types from 1970 to August 2022. During the assessed period, there was exponential growth in the numbers of publications in the field with the most papers published since 1990. Our analysis provides an overview of the published evidence linking dietary patterns, ingredients, and nutrients with cancer and highlights important gaps in the body of literature to guide future research.

#### 4.1.1. Colorectal, Breast, and Liver Cancer Are Prevalent in Nutrition-Related Literature

Our analysis reveals the high prevalence of diet and nutrition research focused on the prevention of and survivorship with colorectal, breast, and liver cancers, and the lower prevalence of dietary research focused on prevention and survivorship in other cancer types, such as prostate, stomach, and lung cancers. Interestingly, the numbers of papers published on breast and colorectal cancer and diet increased exponentially around the mid-1980s and 1990s. Research on colorectal cancer likely increased around this period as it was one of the leading causes of cancer death, along with lung and breast cancer [21], and studies were largely focused on correlations between red meat and fat consumption and increased colorectal cancer risk [22]. The increased number of publications on breast cancer during this time could be tied to the fact that several major breast cancer foundations were started in the 1980s and early 1990s, including but not limited to the Breast Cancer Research Foundation [23] and the Susan G. Komen Breast Cancer Foundation [24]. Additionally, around this time, dietary fat consumption was found to be correlated with the increased incidence of breast cancer [25], which likely spurred increased interest in the relationship between diet and breast cancer. It is likely that research related to diet and lung cancer was scant during this same time as many studies on lung cancer were focused on the role of smoking in its development. Notably, the proportion of diet and nutrition publications across cancer types roughly tracks the leading cancers for the estimated numbers of new cases in the United States, with a few exceptions. For example, prostate (27% in males), breast (31% in females), lung/bronchus (12% in males; 13% in females), and colon/rectum (8% in males and females) are the cancer types with the most new cases per annum and contribute to a significant number of cancer-related deaths [26]. Our analysis highlights gaps in dietary research for cancers with high incidence (i.e., lung and prostate), as well as for less common—but still nutritionally vulnerable—cancer types. For example, cancers of the oral cavity and pharynx and the female genital system often place individuals at a high risk of malnutrition [27] and constitute ~2.8% and ~12.3% of new diagnoses, respectively [26]. However, research on malnutrition amongst patients with these cancer types is scant. Interestingly, our findings differ somewhat from the prior bibliometric analysis [13], in which head and neck cancer and gastric cancers were among the most studied cancer types in the published literature on nutrition and cancer. This discrepancy may be due in part to the limited scope of this prior bibliometric analysis, which focused only on studies published for a recent 10-year period (2011–2021). There is a limited number of studies in the published literature that are sufficiently powered to detect relationships between dietary patterns and components and cancer prevention and survivorship for these less common cancer types [28]. However, the low numbers of publications found for certain cancer types, such as cervical cancer, which is predominantly caused by Human Papilloma Virus [29], may not be due to a lack of research but, rather, the lack of a plausible mechanism to study the impact of diet on cancer development. However, due to the role of diet in the development of obesity, which increases the risk for some cancers, including cervical cancer, diet may still be a significant contributor to the onset and progression of cancers that may be linked to other etiologies [30]. The investigation of nutrition and diet in less prevalent cancer types may require multi-site studies with leadership from many investigators, such as that facilitated by the National Cancer Institute Cohort Consortium. Even among the highly prevalent cancer types, the relationship between diet and cancer risk or outcomes may vary by cancer subtype (i.e., estrogen-responsive versus non-responsive breast cancers). Studies of less prevalent cancer subtypes also benefit from consortium efforts [28,31].

#### 4.1.2. Early Focus on Non-Nutritive Bioactive Compounds and Vitamins with a More Recent Transition to Focus on the Microbiome

Early on, nutrition and cancer research focused on individual macro- and micronutrients and non-nutritive bioactive compounds (i.e., polyphenols and other phytochemicals) for cancer prevention specifically. Epidemiological evidence suggested that diets high in phytochemical- and vitamin-rich fruits and vegetables, particularly rich in vitamins C and E, were associated with decreased cancer risk. Additionally, a seminal article was published by Peto and colleagues, entitled “Can dietary carotene materially reduce human cancer rates?”, which drew more attention to the field [32]. However, when the supplementation of individual nutrients such as alpha-tocopherol, beta-carotene, vitamin C, selenium, or B vitamins was tested in randomized controlled trials, no reduction in cancer risk or cancer-related mortality was observed [33,34,35,36,37] and, in some cases, there was an increase in cancer incidence [38,39,40]. In this example, more recent meta-analyses of this body of work have concluded that β-carotene supplementation does not impact cancer incidence (no harm or benefit), but it may negatively impact lung cancer risk for smokers [41]. This research focus is reflected in our study as the terms “vitamin” and “phytochemical” generated the largest number of articles relative to other bioactive compounds. Additionally, there was increased interest in the role of phytochemicals as chemopreventatives beginning in the 1990s [42], which has continued to this day, despite the aforementioned findings. In nutritional epidemiologic research, traditional dietary assessment methods such as food frequency questionnaires often preclude the ability to investigate some diet exposures, such as probiotics/prebiotics, owing to their lack of inclusion in the measurement instrument, which may also have contributed to our findings. In addition, the role of the gastrointestinal microbiome and the use of prebiotics and probiotics in cancer prevention and treatment has become increasingly researched during the past two decades; specifically, a previous bibliometric analysis of gut microbiome research in cancer reported 10 articles in 2001 and 486 in 2020 [9].

#### 4.1.3. WCRF/AICR Recommendations Have Remained Relatively Constant over Time

The WCRF/AICR recommendations acknowledge the potential role of dietary components or food groups in cancer prevention, are used worldwide, and are also part of the European Code Against Cancer [43]. The initial AICR guidelines of 1984 included recommendations to limit dietary fat, alcohol, and cured and smoked meats while increasing the intake of fruits and vegetables. While the details surrounding these recommendations have changed with active research in this area, certain aspects of these recommendations remain in the 2018 WCRF/AICR guidelines for cancer prevention. For example, current dietary recommendations focus on including whole grains, fruits, vegetables, and legumes as a primary part of the diet, while limiting fast foods, red meat, processed meat, sugary drinks, and alcohol [1]. In this bibliometric analysis, fruits, vegetables, and alcohol were the most studied dietary components. While the number of publications has increased over time for many dietary components, research investigating the link between vegetable consumption and cancer has plateaued over the past twenty years, which is interesting given its inclusion in the 2018 WCRF/AICR guidelines. This bibliometric analysis did not use search terms to separate cancer prevention from treatment. However, the American Society of Clinical Oncology recently published a guideline on exercise, diet, and weight management during cancer treatment that found the relationship between diet and cancer treatment inconclusive due to the paucity of studies in this area [44]. With respect to cancer survivors, an observational study of fruit and vegetable consumption among cancer survivors found 28 studies with an inverse association between vegetable intake and mortality, but this conclusion was only significant for head and neck and ovarian cancer survivors. In other cancers, evidence was insufficient, but associations were positive, highlighting the need for more studies to clarify the results [45]. Although the European Society for Medical Oncology (ESMO) has broad guidelines with respect to the promotion of cancer survivorship, the coverage of diet in this document largely corresponds with that of the aforementioned guidelines and organizations.

#### 4.1.4. Interest Is Increasing in Dietary Patterns over Dietary Content of Foods

It is now generally accepted that dietary patterns are more important in relation to cancer prevention and survivorship than single foods or nutrients [46], an idea supported by the increase in papers on dietary patterns and cancer since 2010. Our analysis revealed that high-fat diets were the most studied dietary pattern in relation to cancer, with a lower proportion of studies focused on overall dietary patterns generally considered detrimental (e.g., western dietary pattern) or beneficial (e.g., healthy or prudent dietary pattern, Mediterranean diet, plant-based diet). This may also be due, in part, to the common use of high-fat diets to induce obesity in animal models of obesity and cancer [47,48,49]. In addition to being the most studied diet with respect to cancer, the number of publications that examined cancer and high-fat diets increased steadily over much of the period analyzed. This finding is likely due to the increase in the percentage of fat in the average American citizen’s diet that occurred around 2010 [50,51]. In this regard, it is surprising that the amount of literature on the intersection of high-fat diets and cancer increased several decades before this actual shift in fat consumption. On the other hand, the numbers of articles related to healthy eating, ketogenic, and western-style diets and cancer began to steadily increase starting in 2010. Furthermore, the focus of the current literature on dietary patterns such as plant-based diets may be due, in part, to the 2018 WCRF/ACS dietary guidelines, which emphasize the importance of a plant-based diet [1,2]. Specifically, these guidelines recommend five portions (400 g or 15 oz) of non-starchy vegetables and fruit each day.

Interestingly, our analysis found that publications using terms related to “plant-based” were uncommon, despite the consumption of fruits and vegetables being a core recommendation from cancer prevention agencies. To date, the scientific evidence does not support any specific plant-based diet over another for the reduction of cancer risk [52,53]. Indeed, the heterogeneity between plant-based dietary patterns (i.e., vegan, vegetarian, Mediterranean, or the New American Plate focus on plants) makes it difficult to perform a direct comparison of particular diets. In a 2019 review of how the Mediterranean diet influences cancer risk, the antioxidants and micronutrients found in fruits and vegetables had an anti-tumorigenic effect on epithelial cancer, digestive, genital, and urinary tract cancer, as well as female breast cancer [54]. However, this analysis only included 53 papers over a ten-year period [54]. Therefore, there is a critical need to expand research on the relationship between dietary patterns and cancer to include other cancer types. Additionally, as mentioned previously, there is a paucity of data on the effects of dietary patterns on cancer treatment [44]. Therefore, future research should investigate how different diet types affect the disease continuum, with a particular focus on randomized controlled trials of diet effects on cancer treatment, to better understand potential diet–cancer links.

Results from our analysis indicate there is a paucity of publications related to caloric restriction or time-restricted feeding and cancer prevention. This finding corresponds to a previous bibliometric analysis that reported only nine published articles for time-restricted eating prior to 2016 [55]. However, our data, similar to previous bibliometric analyses, noted an increase in articles using terms related to fasting or time-restricted eating around the mid-2000s [55,56]. The steady increase in the number of articles related to calorie restriction and time-restricted feeding may be due to the utility of these approaches to manage body weight as a means to prevent cancer [57] or to manage potential interactions of feeding and chemotherapy effectiveness [58]. Because many cancers are obesity-related, including the most common cancers in our analysis—breast and colorectal [59]—and because the worldwide prevalence of obesity is still increasing [60], it is anticipated that the volume of research investigating caloric restriction and fasting in cancer will continue to increase over the coming years. Indeed, there are several ongoing clinical trials to assess the effects of dietary caloric restriction on mortality outcomes in breast, ovarian, and prostate cancer [61,62,63,64].

#### 4.1.5. Nutrition and Cancer Studies Are Represented across the Scientific Spectrum

The network analysis revealed different results based on MeSH keywords versus author-generated keywords, which highlights the importance of conducting literature searches using both term types. Network analyses from MeSH terms uncovered several words related to observational studies (i.e., “risk factors”, “prospective studies”, “case–control studies”), but there were not sufficient articles on clinical trials. This finding is not surprising as many recommendations for diet and nutrition in relation to cancer treatment and survivorship rely on observational data or expert opinion, due to the difficulty in conducting long-term, controlled nutrition trials in these patient populations [65,66]. The author-generated network analysis also illustrated a low amount of research investigating cellular mechanisms together with metabolic diseases or malnutrition. Specifically, topics related to phytochemicals and cancer cell metabolism (cluster 4 of the author-generated network analysis), inflammation, obesity, and metabolic diseases (cluster 2), and malnutrition and outcomes (cluster 3) were poorly linked. While individual articles may not reflect entire funded projects, our analysis suggests a possible lack of transdisciplinary research that bridges the gaps between various focus areas in diet and cancer research (e.g., cell metabolism, patient outcomes, dietary components). Data from the network analyses correspond with our results from the distribution of articles according to cancer type. Specifically, terms related to breast, prostate, and colorectal cancer were among the most prevalent, which highlights the need for more transdisciplinary dietary research in other cancer types.

#### 4.1.6. Comparisons to Other Bibliometric Analyses

There are five other bibliometric analyses examining the relationship between nutrition and cancer. In 2014, 2396 publications on diet and breast cancer were analyzed to assess the number of publications covering these topics and the impact of economic outcomes on national academic productivity [12]. These investigators found an annual, worldwide increase in the number of publications on diet and breast cancer, an observation fully supported by our analysis. Search terms in this analysis were limited to “diet” and “breast cancer”. In 2022 and 2023, four more bibliometric analyses were published, each with a narrower scope than our analysis.

In the first study, 2061 publications were examined to determine the correlation between the gut microbiota and cancer development. The authors of this study incorporated a large diversity of search terms, including terms for specific bacteria and probiotics. Similar to our study, this analysis included multiple search terms for cancer, including neoplasm, tumor, and malignant [9]. In this paper, the researchers found that most research focused on the effect of probiotics in cancer. This conclusion is in contrast to our inference that the prebiotic and probiotic terms generated few publications. However, our study contained over 10,000 publications, and probiotics were mentioned in 701 of these papers. If the February 2022 study had captured all of these in their 2061 results, it would have appeared much more representative of the current scope of research in the field.

A study published in March 2022 that focused on global trends in nutrition in cancer research used solely “nutrition”- and “cancer”-related words to examine publication trends, top published authors and affiliations, and the top ten keywords [11]. As we report here, these authors observed a growing number of publications between 2019 and 2022, with a large body of research on breast cancer. In November of 2022 and February of 2023, two additional bibliometric analyses were published, focusing on the ketogenic and Mediterranean diets, respectively [10,11]. The ketogenic diet paper identified breast and glioma as cancer types commonly studied in the context of this diet, with the numbers of papers on hepatocellular and pancreatic cancers rapidly rising [10]. In the Mediterranean diet study, a review of 1415 articles revealed an upward trend in studies but did not distinguish the number of papers by cancer type [11].

#### 4.1.7. Summary of Gaps in the Literature

Our study reveals several important gaps in the literature, providing opportunities for further investigation. While the annual growth rate of publications in the field is 5.15%, most of these are in breast, colorectal, liver, prostate, and stomach cancer. Additionally, the data suggest that publications in the field of oncology have been rising at a faster rate than those in nutrition, and that research on the combination of nutrition and cancer represents only a small fraction of either of these very large fields. This is consistent for studies focused on overall nutrition and on specific diet types and components. A summary of the specific combinations of cancer type and diet pattern/component with very few or no publications is included in Supplemental Table S5. Overall, opportunities remain to study the impact of nutrition on ovarian, bladder, brain, endometrial, skin, cervical, kidney, and gallbladder cancer and leukemia. There is also a gap in the literature regarding certain diet patterns, including DASH, gluten-free, low-fat, time-restricted eating/feeding, and paleolithic diets, both in cancer in general and in specific cancer types. While dietary patterns are likely more important than single dietary components, future studies that include an evaluation of the role of refined grains, chocolate, plant protein, and fried food are limited and may provide additional insights, especially in relation to cancers of the brain, cervix, gallbladder, or kidney.

### 4.2. Strengths and Limitations

Our analysis provides a comprehensive overview of the entire scope of the diet and cancer research field and, as such, the findings are available to help guide future research in the field. Strengths of the analysis are the use of systematic methods to capture all relevant papers and the implementation of a team science approach that included consulting the literature, refining the search strategy through multiple rounds of discussion, and pilot testing before finalizing the search terms. We were similarly systematic in the identified paper screening procedure. As such, we are confident that the analytical approach has identified the majority of diet and cancer research papers related to nutritional effects on cancer types, including those that are often not included in more focused reviews and analyses. Additional strengths of our analysis are the inclusion of articles from all languages and additional secondary searches of terms related to specific dietary patterns and components.

Despite these many strengths, all studies have limitations. Firstly, as is the case for all bibliometric reviews, our results were highly dependent on the search terms used. For our specific review, it was difficult to discern the specific type of diet within the broader dietary MeSH terms. As with any bibliometric review, the MeSH and search terms for titles and abstracts may have identified other studies that were not related to nutrition or cancer. As such, not all the retrieved studies may have been directly related to cancer research. It is also possible that the analysis missed some papers that did not include informative keywords in the title or abstract. Additionally, our search terms retrieved a small number of irrelevant articles that may have been included in the data pool. Further, it was very difficult to differentiate between, for example, human and animal or cell culture studies and to determine whether a study specifically addressed cancer risk, cancer treatment, or survivorship, since these types of studies may refer to each other in the background or discussion section of the abstract. However, these are limitations common to all bibliometric reviews, which are intended to provide a general overview of publication trends. We believe that our highly selective secondary keyword searches were able to sufficiently capture general trends in publications for this research field. Lastly, publications indexed in other medical and scientific databases outside of PubMed were not included in this analysis. This is likely a minor limitation, as we contend that the majority of diet- and cancer-related research is accurately indexed in PubMed.

## 5. Conclusions

This is the first comprehensive bibliometric analysis that describes the scientific literature on dietary patterns, components, and nutrients in relation to effects on a variety of cancer types. Our results reveal an extensive body of literature published on diet in breast, colorectal, and liver cancer. However, relatively little research has examined the relationship between diet and the other cancer types included in this analysis, including some prevalent cancer types, such as prostate, endometrial, and lung. While the WCRF/AICR recommendations have remained relatively constant over time, there has been a shift in the literature over the last 20 years from studying non-nutritive bioactive components to studying specific diet types (i.e., Mediterranean, vegan, calorie-restricted) and dietary patterns. Our findings highlight significant gaps in the diet and cancer research field, which should be addressed. The data provide a platform from which to consider the reexamination of the lifestyle and dietary guidelines for cancer prevention. Overall, this analysis serves as a valuable resource to guide future studies of a variety of cancer types and diet patterns, components, and nutrients and establish meaningful connections between nutrition and cancer risk, treatment, and survivorship.

## Data Availability

Data used for these analyses will be available through the University of Michigan Deep Blue Data Repository. The DOI will be available once deposited.

## Supplementary Materials

The following supporting information can be downloaded at: https://www.mdpi.com/article/10.3390/cancers15153761/s1, Figure S1: MeSH generated key words, Figure S2: Author-generated keywords; Table S1: Search terms within the main query used to identify specific cancer types; Table S2: Search terms within the main query used to identify dietary energy modulation or pattern; Table S3: Search terms within the main query used to identify dietary components; Table S4: Search terms within the main query used to identify dietary nutrients, supplements, and vitamins; Table S5: Summary of the relevant gaps in the diet and cancer literature.
