# Supplementary material for: Trends in Diet and Cancer Research: A Bibliometric and Visualization Analysis
Source: Cancers (Basel). 2023 Jul 25;15(15):3761. doi: 10.3390/cancers15153761 (PMC10417030; doi:10.3390/cancers15153761)
Supplement: Supplementary file 1 [file cancers-15-03761-s001.zip › cancers-2442443-supplementary.pdf]

Cluster 1 - Males, animals, cellular mechanisms

Keyword	Occurrences
male	43990
animals	32668
mice	11506
rats	11445
liver	5485
obesity	4782
carcinogens	4494
colonic neoplasms	4427
prostatic neoplasms	4293
body weight	4247
dietary supplements	4213
dietary fats	4121
antioxidants	4016
time factors	3997
liver neoplasms	3831
disease models, animal	3525
mice, inbred c57bl	3499
inflammation	3442
adenocarcinoma	3240

Cluster 2 - Humans, lifestyle, risk

Keyword	Occurrences
humans	70556
diet	19063
neoplasms	14054
risk factors	13982
prospective studies	6688
breast neoplasms	6566
case-control studies	5311
colorectal neoplasms	4713
surveys and questionnaires	4542
cohort studies	3956
incidence	3791
feeding behavior	3442
united states	3393
body mass index	3326
smoking	3292
life style	3066
energy intake	2992

Cluster 3 – Female, age groups

Keyword	Occurrences
female	43596
middle aged	26705
aged	21477
adult	20199
aged, 80 and over	5964
adolescent	4831
nutritional status	4774
retrospective studies	4433
treatment outcome	4114
follow-up studies	3742
stomach neoplasms	3681
prognosis	3665
child	3336
young adult	3217

Figure S1. MeSH generated key words.

Cluster 1 – Inflammation, obesity, metabolic conditions

Keyword	Occurrences
inflammation	1887
obesity	1639
colorectal cancer	1335
oxidative stress	812
apoptosis	709
hepatocellular carcinoma	499
diabetes	442
insulin resistance	424
gut microbiota	420
colon cancer	397
chemoprevention	363
metabolism	328
high-fat diet	327
metabolic syndrome	321
aging	258
epigenetics	241

Cluster 2 - Nutrition status, treatment, treatment outcomes

Keyword	Occurrences
nutrition	1460
gastric cancer	659
malnutrition	642
quality of life	506
prognosis	504
chemotherapy	459
head and neck cancer	397
survival	380
sarcopenia	374
nutritional status	357
pancreatic cancer	345
cachexia	286
weight loss	260
radiotherapy	243
body mass index	211

Cluster 3 - General lifestyle and study design terms

Keyword	Occurrences
diet	1655
breast cancer	1327
mortality	643
prostate cancer	587
epidemiology	570
physical activity	488
meta-analysis	456
exercise	375
risk factors	367
cardiovascular disease	356
prevention	293
lifestyle	286
mediterranean diet	286

Cluster 4 – General field terms

Keyword	Occurrences
cancer	2662
neoplasms	292
health	230
biology	226
diseases	222
physiology	209

Figure S2. Author-generated keywords.

Supplemental Table S1. Search terms within the main query used to identify specific cancer types			
**Cancer Type**	**Term(s) Queried in MeSH Field**	**Terms Queried in Abstracts** (" | " means "OR"; "." means any character; " * " means includes letters after stem word)	**# of Records Returned**
Bladder	Urinary Bladder Neoplasms	bladder cancer.|cancer of the bladder|bladder tumor|bladder tumour	1451
Brain / Glioblastoma	Glioblastoma<br>Brain Neoplasms<br>Central Nervous System Neoplasms	glioblastoma.|brain cancer.|brain tumor.|brain tumour.|brain neoplasm.|meningioma|glioma|atrocytic tumo*|astrocytoma|ependymoma|meningeal tumo*|oligodendroglioma	879
Breast	Breast Neoplasms<br>Mammary Neoplasms, Animal	breast cancer.|breast tumor.|breast tumour.|breast neoplas.|mammary cancer.|mammary tumor.|mammary tumour|mammary neoplas.|ductal carcinoma|lobular carcinoma|DCIS	10373
Cervical	Uterine Cervical Neoplasms<br>Cervical Intraepithelial Neoplasia	cervical cancer.|cancer of the cervix|cervical neoplas*|cervical intraepithelial neoplas*|cervix dysplasia	1333
Colorectal	Colorectal Neoplasms<br>Intestinal Polyps	colorectal cancer.|colorectal tumor.|colorectal tumour.|colon cancer.|rectal cancer.	11942
Endometrial	Endometrial Neoplasms	endometrial cancer.|endometrial tumor.|endometrial tumour.|endometrial carcinoma	1609
Esophageal cancer	Esophageal Neoplasms	esophageal cancer.|esophageal tumor.|tumor of the esophagus|esophag. cancer	2881
Gallbladder	Gallbladder Neoplasms<br>Biliary Tract Neoplasms	Gallbladder cancer.|cancer.of the gallbladder|gallbladder neoplasm|gallbladder carcinoma|gallbladder tumo*	387
Kidney	Kidney Neoplasms<br>Urethral Neoplasms	kidney cancer.|renal cancer.|kidney tumo*|renal tumo*|Wilms tumo*|grawitz tumo*|hypernephroma|renal cell carcinoma|transitional cell carcinoma|urothelial cancer.|urothelial carcinoma	1097
Liver	Liver Neoplasms<br>Liver Neoplasms, Experimental	liver cancer.| hepatic cancer.|liver tumo*|hepatic tumo*|hepatocarcinoma|hepatocellular carcinoma|hepatoma|cholangiocarcinoma	7178
Lung	Lung Neoplasms	lung cancer.|lung tumo*|pulmonary tumor|pulmonary tumour|small cell carcinoma of the lung	4014
Leukemia	Leukemia	leukemia.	1872
Mouth, Nasopharynx, & Larynx	Head and Neck Neoplasms| Laryngeal Neoplasms| Pharyngeal Neoplasms| Mouth Neoplasms| Tongue Neoplasms| Oropharyngeal Neoplasms| Hypopharyngeal Neoplasms| Oral Leukoplakia	mouth cancer.|oral cancer.| oral tumo*|nasopharyngeal cancer.|nasopharyngeal tumo*|pharyn. tumo*|larynx tumo*	3282
Ovarian	Ovarian Neoplasms	ovarian cancer.|ovarian tumo*|tumor of the ovar.|tumour of the ovar.| Endometrioid carcinoma| cystoadenoma.| cystoadenocarcinoma.| Androblastoma.| arrhenoblastoma*| sertoli.leydig| Brenner| granulosa cell tumo*| luteoma*| luteinoma*	1242
Pancreatic	Pancreatic Neoplasms	pancreatic neoplas*|pancreas neoplas*|pancreatic cancer*|pancreas cancer*|pancreatic carcin*|pancreas carcin*|pancreatic tumo*|pancreas tumo*|pancreatic metasta*|pancreas metasta*|pancreatic malign*|pancreas malign*|pancreatic adenocarinoma*|pancreas adenocarcinoma*	2412
Prostate	Prostatic Neoplasms	prostate cancer.|prostate tumo*|prostatic tumo*|prostatic adenocarcinoma|prostatic carcinoma|prostatic cancer.|prostate neoplasm*|prostatic neoplasm*	5321
Skin	Skin Neoplasms	skin cancer.|skin tumo*| melanoma. | basal.cell.carcinoma.| merkel.cell.carcinoma| cutaneous.T.cell.lymphoma.	1751
Stomach	Stomach Neoplasms	stomach cancer.|stomach tumor.|stomach tumour.|gastric cancer.| gastric carcinoma.|gastrointestinal stromal tumo*	4765

Supplemental Table S2. Search terms within the main query used to identify dietary energy modulation or pattern		
**Diet MeSH term**	**Terms Queried in Abstracts (** " | " means "OR"; "." means any character; " * " means includes letters after stem word**)**	**# of Records Returned**
**Dietary energy modulation**		
Caloric Restriction	caloric restriction| energy.restrict.| calorically.restricted| energy.restricted| low.calorie| low.caloric| low.energy.diet| hypocaloric	965
Ketogenic or low-carbohydrate	ketogenic| keto diet| ketosis| low.carb*| carbohydrate.restricted| reduced.carbohydrate| high.protein.diet| diet high in protein| Atkins diet	1312
High Fat	high.fat| diet. high.in.fat|HF.diet|HFD	4936
Low Fat	low fat diet.|low in fat|LF.diet|LFD	355
**Diet pattern**		
DASH Diet	dash diet | dietary approaches to stop hypertension	137
Gluten Free	gluten.free| gluten-free| gluten.restrict*	307
Healthy Diet Patterns	healty.eating.index| healthy.diet| healthy.lifestyle.index| prudent.diet.| diet.quality| diet.score	1460
Mediterranean Diet	mediterranean.diet| mediterranean eating| mediterranean.style.diet| meddiet	1045
Paleolithic	paleo.diet.|paleolithic.diet*	17
Plant Based	vegetarian*|vegan|macrobiotic|plant.based	919
Time Restricted	alternate.day.fasting|time.restricted|fasting.mimicking|intermittent.energy.restriction|intermittent.calorie.restriction|intermittent.caloric.restriction|intermittent.feeding	95
Western Diet	western.diet.| western.style.diet.| western.pattern.diet.| american.diet| cafeteria.diet| CAF.diet| obesogenic diet	975

Supplemental Table S3. Search terms within the main query used to identify dietary components		
**Diet Term**	**Search Term** (" | " means "OR"; "." means any character; " * " means includes letters after stem word)	**# of Records Returned**
Alcohol	alcohol|wine|liquor|beer	7243
Carbohydrates	dietary carbohydrate.|carbohydrate|bread|pasta	2965
Chocolate	chocolate|cocoa	154
Coffee/Tea	coffee.tea	2885
Dairy	dairy products|milk|cheese|cultured milk product.| yogurt|yoghurt	2224
Dietary Fat/Oil	dietary fat|butter|ghee|cholesterol|oil|margarine	4978
Dietary Fiber	fibre|fiber|wheat bran.|roughage.|resistant starch	3773
Fish/Seafood	fish|seafood|shellfish	3453
Fried Food	fried.food.	114
Fruit	fruit|berry|berries|citrus|melon.|pome.	8203
Genetically Modified	genetically.modified food|genetically.modified organism|GMF|GMO	376
Honey	honey	114
Nuts	nuts|seeds	946
Organic Food	organic	870
Plant Protein	plant.protein|plant.derived.protein|plant.based.protein|pea.protein|vegetable.protein	150
Poultry	poultry|chicken|turkey|duck|goose	847
Processed Food	processed.food|fast food|convenience.food.|ready.to.eat|ready.prepared.food.	301
Red Meat	red.meat|beef|lamb|goat|veal	1531
Refined Grain	refined.grain|white.flour|refined.carbohydrate	256
Saturated Fat	saturated.fat|saturated.dietary.fat	1318
Soy	soy|natto|tempeh|tofu|bean.curd|miso	1162
Spices	spice.|seasoning	467
Sugar	dietary sugar|dietary sucrose|table sugar|white sugar|added sugar.|corn syrup|sugar.sweetened.beverage.|soda |cola	333
Unsaturated Fat	unsaturated.dietary.fat.|dietary.oil.|oil|oils|dietary.omega.3|omega.3.fatty.acid.|MUFA|PUFA	3940
Vegetables	vegetable.	6714
Whole Grain	whole.grain|wholegrain|whole.wheat|whole.meal|unrefined grain.|coarse.grain|unprocessed.grain	731

Supplemental Table S4. Search terms within the main query used to identify diet nutrients, supplements, and vitamins		
**Diet Term**	**Search Term** (" | " means "OR"; "." means any character; " * " means includes letters after stem word)	**# of Records Returned**
Dietary Supplements	dietary.supplement*|food.supplement*|nutraceutical. |neutraceutical.|herbal.supplement.	3104
Micronutrients	micronutrient.	1222
Minerals	mineral.	1445
Phytochemicals	dietary.phytochemical.|plant.bioactive.compound.|plant.biologically.active.compound.|plant.derived.chemical.|plant.derived.chemicals|plant.bioactive.coumpound.|phytochemical.|phytonutrient.|plant.derived.compound.|flavanol.|flavonoid.|polyphenol.|carotenoid||alkaloid|amines|cyanogenic.glycosides|diterpenes|glucosinolate.|monoterpenes|non-protein amino acids|phenylpropanes|polyacetylenes|polyketides|sesquiterpenes|tetraterpenes|triterpenes|saponins|plant steroids	7801
Prebiotics	prebiotic.	378
Probiotics	probiotic.	751
Synbiotics	synbiotic.	109
Vitamins	vitamin.	7240

Supplemental Table S5. Summary of the relevant gaps in the diet and cancer literature

Diet Type

Cancer Type	Paleolithic	Time Restructed	DASH	Gluten Free	Low Fat	Western-Style	Calorie Restricion	Plant Based	Healthy Eating	Mediterranean Diet	Ketogenic Diet
Bladder	X	X	X	X	X	X	X				
Brain	X	X	X	X	X	X		X	X	X	
Breast	X			X							
Cervical	X	X	X	X	X	X	X	X	X	X	X
Colorectal	X										
Endometrial	X	X	X	X	X						
Esophageal	X	X	X		X	X	X				
Gallbladder	X	X	X	X	X	X	X	X	X	X	
Kidney	X	X	X	X	X	X		X	X	X	
Leukemia	X	X	X		X	X		X			
Liver	X	X	X	X							
Lung	X	X		X							
Mouth/Nasopharynx	X	X	X	X	X	X	X				
Ovarian	X	X	X	X							
Pancreatic	X	X	X								
Prostate	X	X	X	X							
Skin	X	X	X	X							
Stomach	X	X	X		X						

Diet Component

Cancer Type	Honey	Chocolate	GMO	Plant Protein	Processed	Refined Grain	Sugar	Fried Food	Spices	Whole Grain	Organic	Saturated Fat	Soy	Nuts	Poultry	Red Meat	Dairy	Coffee	Fish	Unsaturated Fat
Bladder	X	X	X	X	X	X	X	X	X	X		X		X	X					
Brain	X	X	X	X	X	X	X	X	X	X	X	X	X	X	X		X			
Breast	X		X																	
Cervical	X	X	X	X	X	X	X	X	X	X	X	X	X	X	X	X		X	X	X
Colorectal	X			X																
Endometrial	X	X	X	X	X	X	X	X	X		X				X					
Esophageal	X	X	X	X	X	X	X		X	X	X	X	X							
Gallbladder	X	X	X	X	X	X	X	X	X	X	X	X	X	X	X	X	X			
Kidney	X	X	X	X	X	X	X	X	X	X	X	X	X	X						
Leukemia	X	X	X	X	X	X	X	X		X	X	X	X	X						
Liver	X	X	X	X	X	X	X	X		X										
Lung	X	X	X	X	X	X	X			X										
Mouth/Nasopharynx	X	X	X	X	X	X	X			X	X	X	X	X						
Ovarian	X	X		X	X	X	X	X	X		X			X						
Pancreatic	X	X	X	X	X	X	X	X	X	X										
Prostate	X	X	X		X			X	X											
Skin	X	X	X	X	X	X	X	X	X	X		X	X		X	X				
Stomach	X	X	X	X	X	X	X	X												

Micronutrients

Cancer Type	Synbiotics	Prebiotics	Probiotics	Minerals	Micronutrients	Phytochemicals	Supplements	Vitamins
Bladder	X	X	X					
Brain	X	X	X	X	X			
Breast	X							
Cervical	X	X	X	X				
Colorectal	X							
Endometrial	X	X	X					
Esophageal	X	X	X					
Gallbladder	X	X	X	X	X	X	X	X
Kidney	X	X	X					
Leukemia	X	X	X					
Liver	X	X	X					
Lung	X	X	X					
Mouth/Nasopharynx	X	X	X					
Ovarian	X	X	X	X				
Pancreatic	X	X	X					
Prostate	X	X	X					
Skin	X	X	X					
Stomach	X	X						

X indicates a low number or no papers identified in our analysis for this combination of cancer and diet term
